# Supplementary material for: Caesarean section delivery and childhood obesity in a British longitudinal cohort study
Source: PLoS One. 2019 Oct 30;14(10):e0223856. doi: 10.1371/journal.pone.0223856 (PMC6821069; doi:10.1371/journal.pone.0223856)
Supplement: S1 Table — (PDF) [file pone.0223856.s001.pdf]

S1 Table. International Obesity Task Force classification of body mass index from age three to fourteen and body fat% at age seven and fourteen.

Characteristic	Overall n (%)	Normal vaginal delivery n (%)	Assisted vaginal delivery [a] n (%)	Planned Caesarean section n (%)	Emergency Caesarean section n (%)
Body mass index (kg/m^2) at 3 years*					
Thin	178 (1.0)	110 (0.9)	17 (1.0)	19 (1.1)	32 (1.5)
Normal	11953 (66.0)	8,303 (66.1)	1114 (66.4)	1111 (66.6)	1,425 (64.7)
Overweight	947 (5.2)	622 (4.9)	100 (6.0)	98 (5.9)	127 (5.8)
Obese	330 (1.8)	217 (1.7)	28 (1.7)	35 (2.1)	50 (2.3)
Missing	4708 (26.0)	3315 (26.4)	418 (24.9)	406 (24.3)	569 (25.8)
Body mass index (kg/m^2) at 3 years					
Thin	706 (3.9)	483 (3.8)	50 (3.0)	67 (4.0)	106 (4.8)
Normal	9568 (52.8)	6,675 (53.1)	890 (53.1)	890 (53.3)	1,113 (50.5)
Overweight	2376 (13.1)	1,590 (12.7)	245 (14.6)	226 (13.5)	315 (14.3)
Obese	758 (4.2)	498 (4.0)	73 (4.4)	84 (5.0)	103 (4.7)
Missing	5,414 (29.9)	3,804 (30.3)	469 (28.0)	469 (28.1)	672 (30.5)
Body mass index (kg/m^2) at 5 years					
Thin	699 (3.9)	476 (3.8)	66 (3.9)	60 (3.6)	97 (4.4)
Normal	10313 (56.9)	7,224 (57.5)	973 (58.0)	921 (55.2)	1,195 (54.2)
Overweight	2266 (12.5)	1,500 (11.9)	228 (13.6)	248 (14.9)	290 (13.2)
Obese	834 (4.6)	557 (4.4)	77 (4.6)	84 (5.0)	116 (5.3)
Missing	3,286 (26.0)	399 (26.1)	416 (23.8)	602 (24.9)	3,286 (27.3)
Body mass index (kg/m^2) at 7 years					
Thin	750 (4.1)	526 (4.2)	63 (3.8)	60 (3.6)	101 (4.6)
Normal	9282 (51.2)	6,475 (51.5)	900 (53.7)	824 (49.4)	1,083 (49.2)
Overweight	1966 (10.9)	1,321 (10.5)	174 (10.4)	217 (13.0)	254 (11.5)
Obese	873 (4.8)	575 (4.6)	77 (4.6)	97 (5.8)	124 (5.6)
Missing	5,995 (33.1)	4,196 (33.4)	526 (31.4)	531 (31.8)	742 (33.7)
Body mass index (kg/m^2) at 11 years					
Thin	722 (4.0)	492 (3.9)	72 (4.3)	69 (4.1)	89 (4.0)
Normal	7946 (43.9)	5,546 (44.1)	789 (47.0)	671 (40.2)	940 (42.7)
Overweight	2607 (14.4)	1,767 (14.1)	220 (13.1)	268 (16.1)	352 (16.0)
Obese	870 (4.8)	596 (4.7)	64 (3.8)	99 (5.9)	111 (5.0)
Missing	6,693 (36.9)	4,658 (37.1)	604 (36.0)	631 (37.8)	800 (36.3)
Body mass index (kg/m^2) at 14 years					
Thin	645 (3.6)	463 (3.7)	61 (3.6)	55 (3.3)	66 (3.0)
Normal	6815 (37.6)	4,728 (37.6)	672 (40.1)	602 (36.1)	813 (36.9)
Overweight	2475 (13.7)	1,411 (11.2)	165 (9.8)	602 (36.1)	297 (13.5)
Obese	797 (4.4)	545 (4.3)	59 (3.5)	81 (4.9)	112 (5.1)
Missing	8,029 (44.3)	5,883 (46.8)	781 (46.6)	384 (23.0)	981 (44.5)
Body fat (%), median IQR at age 7	20.1 (17.5-23.8)	20.1 (17.5-23.7)	20 (17.4-23.4)	20.6 (17.8-24.2)	20.3 (17.5-24.2)
Missing	5435 (30.0)	3801 (30.2)	486 (29.0)	488 (29.2)	660 (30.0)
Body fat (%), median IQR at age 14	21.7 (14.4-28.4)	22 (14.4-28.4)	20 (13.8-26.8)	22.6 (15.3-29.5)	21.1 (14.4-28.8)
Missing	7898 (43.6)	5,510 (43.8)	733 (43.7)	722 (43.3)	933 (42.4)

*World Health Organization z-scores

Masukume G, Khashan AS, Morton SMB, Baker PN, Kenny LC, McCarthy FP. Caesarean section Delivery and Childhood Obesity in a British Longitudinal Cohort Study. *PLoS One*. 2019. DOI: 10.1371/journal.pone.0223856
