# Supplementary material for: Caesarean section delivery and childhood obesity in a British longitudinal cohort study
Source: PLoS One. 2019 Oct 30;14(10):e0223856. doi: 10.1371/journal.pone.0223856 (PMC6821069; doi:10.1371/journal.pone.0223856)
Supplement: S2 Table — (PDF) [file pone.0223856.s002.pdf]

S2 Table. Missing data for body mass index at age two years.

Characteristic	Body mass index missing (n %) n= 5487	Body mass index not missing (n %) n=12772	p-value[a]
Maternal age (years), median IQR[b]	27 (22-32)	29 (25-33)	< 0.001
Ethnicity			< 0.001
European	4325 (28.5)	10,855 (71.5)	
Asian	695 (36.4)	1,216 (63.6)	
African	262 (39.5)	402 (60.5)	
Mixed	79 (42.5)	107 (57.5)	
Any other background	68 (46.6)	78 (53.4)	
Highest education			< 0.001
GCSE grades D-G	671 (34.5)	1,273 (65.5)	
O level / GCSE grades A-C	1,757 (29.1)	4,290 (70.9)	
A / AS / S levels	421 (25.0)	1,266 (75.0)	
Diplomas in higher education	372 (24.6)	1,139 (75.4)	
First degree	411 (18.4)	1,818 (81.6)	
Higher degree	132 (21.9)	472 (78.2)	
Other academic qualifications (including overseas)	202 (38.4)	324 (61.6)	
None of these qualifications	1,441 (40.9)	2,080 (59.1)	
Total net couple income (UK pounds)			< 0.001
0-10399	710 (38.2)	1,148 (61.8)	
10400-15599	837 (33.2)	1,685 (66.8)	
15600-19799	706 (27.9)	1,827 (72.2)	
20800-30199	722 (22.7)	2,463 (77.3)	
31200-80000+	642 (20.1)	2,556 (80.0)	
Not applicable	1,387 (39.4)	2,138 (60.7)	
Don't know	292 (31.7)	629 (68.3)	
Refused	139 (37.2)	235 (62.8)	
Marital status			< 0.001
Legally separated	179 (34.7)	337 (65.3)	
Married, 1st and only marriage	2,675 (26.7)	7,341 (73.3)	
Remarried, 2nd or later marriage	176 (24.1)	554 (75.9)	
Single never married	2,179 (35.7)	3,921 (64.3)	
Divorced	211 (29.4)	508 (70.7)	
Widowed	13 (39.4)	20 (60.6)	
Body mass index (kg/m^2) pre-pregnancy, median IQR[b]	22.5 (20.4-25.4)	22.8 (20.8-25.8)	< 0.001
Smoking during pregnancy			< 0.001
Non-smoker	3,744 (29.0)	9,183 (71.0)	
Gave up	714 (31.1)	1,584 (68.9)	
Smoker	971 (33.8)	1,906 (66.3)	
Diabetes mellitus			0.890

Masukume G, Khashan AS, Morton SMB, Baker PN, Kenny LC, McCarthy FP. Caesarean section Delivery and Childhood Obesity in a British Longitudinal Cohort Study. *PLoS One*. 2019. DOI: 10.1371/journal.pone.0223856

Any kind of diabetes mellitus	95 (30.4)	218 (69.7)	
No diabetes mellitus	5,339 (30.0)	12,463 (70.0)	
Number of other children – ‘parity’			
1	5,210 (29.8)	12,264 (70.2)	0.021
2	164 (34.9)	306 (65.1)	
3	59 (35.1)	109 (64.9)	
Sex			< 0.001
Boy	2,922 (31.4)	6,400 (68.7)	
Girl	2,513 (28.6)	6,281 (71.4)	
Gestational age (weeks)			0.001
Preterm (< 37)	562 (32.9)	1,146 (67.1)	
Term (37-41)	4,720 (29.5)	11,272 (70.5)	
Postterm (> 42)	84 (37.3)	141 (62.7)	
Birth weight (kg), median IQR[b]	3.35 (3-3.69)	3.4 (3.03-3.74)	< 0.001

IQR – Interquartile range, BMI – Body mass index, SD – standard deviation, UK – United Kingdom

[a] Pearson’s χ^2 test or Fisher’s exact

[b] Mann-Whitney test
