# Supplementary material for: Caesarean section delivery and childhood obesity in a British longitudinal cohort study
Source: PLoS One. 2019 Oct 30;14(10):e0223856. doi: 10.1371/journal.pone.0223856 (PMC6821069; doi:10.1371/journal.pone.0223856)
Supplement: S3 Table — (PDF) [file pone.0223856.s003.pdf]

S3 Table. Mode of delivery and BMI category transition between ages three and five.

Transition (remained normal – base outcome)	**RRR (95% CI)**	**p-value**	**AdjRRR (95% CI)****	**p-value**
Remained obese				
Normal vaginal delivery	reference		reference	
Assisted vaginal delivery	1.09 (0.71; 1.67)	0.695	1.16 (0.74; 1.85)	0.507
Planned Caesarean	1.20 (0.79; 1.81)	0.394	0.94 (0.59; 1.49)	0.780
Emergency Caesarean	1.22 (0.83; 1.79)	0.317	1.09 (0.70; 1.71)	0.698
Became obese				
Normal vaginal delivery	reference		reference	
Assisted vaginal delivery	0.92 (0.65; 1.30)	0.638	1.11 (0.77; 1.59)	0.572
Planned Caesarean	1.13 (0.82; 1.56)	0.444	0.96 (0.67; 1.38)	0.836
Emergency Caesarean	1.35 (1.02; 1.79)	0.035	1.34 (0.98; 1.82)	0.066
Became non obese				
Normal vaginal delivery	reference		reference	
Assisted vaginal delivery	1.18 (0.81; 1.72)	0.387	1.20 (0.81; 1.78)	0.362
Planned Caesarean	1.06 (0.71; 1.57)	0.787	0.81 (0.51; 1.27)	0.350
Emergency Caesarean	1.03 (0.71; 1.50)	0.872	0.92 (0.61; 1.38)	0.688
Any other transition				
Normal vaginal delivery	reference		reference	
Assisted vaginal delivery	1.04 (0.91; 1.18)	0.579	1.10 (0.97; 1.26)	0.143
Planned Caesarean	0.98 (0.86; 1.12)	0.761	1.01 (0.88; 1.16)	0.921
Emergency Caesarean	1.11 (0.99; 1.25)	0.083	1.11 (0.99; 1.27)	0.082

N for adjusted model = 11,421. Multinomial logistic regression. BMI – Body mass index, RRR (Relative Risk Ratio), CI (Confidence intervals), Adj (Adjusted).
**Adjusted for maternal age, ethnicity, education, marital status, couple income, infant sex, birth weight, smoking, gestational age, diabetes mellitus, parity, pre-pregnancy BMI (Non-macrosomic infants).

Masukume G, Khashan AS, Morton SMB, Baker PN, Kenny LC, McCarthy FP. Caesarean section Delivery and Childhood Obesity in a British Longitudinal Cohort Study. *PLoS One*. 2019. DOI: 10.1371/journal.pone.0223856
