# Supplementary material for: Caesarean section delivery and childhood obesity in a British longitudinal cohort study
Source: PLoS One. 2019 Oct 30;14(10):e0223856. doi: 10.1371/journal.pone.0223856 (PMC6821069; doi:10.1371/journal.pone.0223856)
Supplement: S4 Table — Imputed pre-pregnancy BMI and childhood body fat percent. (PDF) [file pone.0223856.s004.pdf]

S4 Table. Mode of delivery and body fat percent at seven and fourteen years. Imputed pre-pregnancy BMI and childhood body fat percent.

Delivery mode	Coef. (95% CI)	p-value	AdjCoef. (95% CI)**	p-value
Normal vaginal delivery	reference		reference	
Assisted vaginal	-0.16 (-0.50; 0.19)	0.370	0.05 (-0.29; 0.39)	0.781
Planned Caesarean	0.44 (0.09; 0.79)	0.014	0.15 (-0.21; 0.51)	0.412
Emergency Caesarean	0.31 (-0.00; 0.61)	0.053	0.21 (-0.11; 0.53)	0.199
**Delivery mode**	**Coef. (95% CI)**	**p-value**	**AdjCoef. (95% CI)****	**p-value**
Normal vaginal delivery	reference		reference	
Assisted vaginal	-1.27 (-1.87; -0.66)	0.000	-0.41 (-0.96; 0.13)	0.135
Planned Caesarean	0.49 (-0.13; 1.11)	0.120	0.00 (-0.56; 0.57)	0.988
Emergency Caesarean	-0.05 (-0.62; 0.52)	0.861	0.09 (-0.46; 0.63)	0.755

N for adjusted model = 14,595 and 14,595 at age seven and fourteen respectively. Linear regression. BMI – Body mass index, Coef (Coefficient), CI (Confidence intervals), Adj (Adjusted).
**Adjusted for maternal age, ethnicity, education, marital status, couple income, infant sex, birth weight, smoking, gestational age, diabetes mellitus, parity, pre-pregnancy BMI (Non-macrosomic infants).

Masukume G, Khashan AS, Morton SMB, Baker PN, Kenny LC, McCarthy FP. Caesarean section Delivery and Childhood Obesity in a British Longitudinal Cohort Study. *PLoS One*. 2019. DOI: 10.1371/journal.pone.0223856
