# Supplementary material for: Caesarean section delivery and childhood obesity in a British longitudinal cohort study
Source: PLoS One. 2019 Oct 30;14(10):e0223856. doi: 10.1371/journal.pone.0223856 (PMC6821069; doi:10.1371/journal.pone.0223856)
Supplement: S5 Table — (PDF) [file pone.0223856.s005.pdf]

S5 Table. Mode of birth and body mass index for infants with mothers > 35 years old.

BMI		**Coef (95% CI)**	**p-value**	**AdjCoef (95% CI)****	**p-value**
	Normal vaginal	reference		reference	
	Assisted vaginal	0.03 (-0.27; 0.33)	0.857	0.08 (-0.22; 0.38)	0.606
	Planned Caesarean	0.06 (-0.18; 0.29)	0.624	-0.06 (-0.29; 0.18)	0.635
	Emergency Caesarean	-0.07 (-0.31; 0.18)	0.589	-0.21 (-0.46; 0.04)	0.105

Time points for adjusted model = 6,195. Mixed-effects linear regression. BMI – Body mass index, Coef (Coefficient), CI (Confidence intervals), Adj (Adjusted).
**Adjusted for maternal age, ethnicity, education, marital status, couple income, infant sex, birth weight, smoking, gestational age, diabetes mellitus, parity, pre-pregnancy BMI (Non-macrosomic infants).

Masukume G, Khashan AS, Morton SMB, Baker PN, Kenny LC, McCarthy FP. Caesarean section Delivery and Childhood Obesity in a British Longitudinal Cohort Study. *PLoS One*. 2019. DOI: 10.1371/journal.pone.0223856
