# Supplementary material for: Caesarean section delivery and childhood obesity in a British longitudinal cohort study
Source: PLoS One. 2019 Oct 30;14(10):e0223856. doi: 10.1371/journal.pone.0223856 (PMC6821069; doi:10.1371/journal.pone.0223856)
Supplement: S6 Table — (PDF) [file pone.0223856.s006.pdf]

S6 Table. Mode of birth and body mass index for infants born pre-term.

BMI		Coef (95% CI)	p-value	AdjCoef (95% CI)**	p-value
	Normal vaginal	reference		reference	
	Assisted vaginal	0.15 (-0.25; 0.55)	0.453	0.12 (-0.28; 0.53)	0.552
	Planned Caesarean	0.24 (-0.09; 0.57)	0.147	-0.04 (-0.39; 0.30)	0.816
	Emergency Caesarean	-0.33 (-0.55; -0.11)	0.003	-0.12 (-0.36; 0.12)	0.331

Time points for adjusted model = 5,161. Mixed-effects linear regression. BMI – Body mass index, Coef (Coefficient), CI (Confidence intervals), Adj (Adjusted).
**Adjusted for maternal age, ethnicity, education, marital status, couple income, infant sex, birth weight, smoking, gestational age – omitted because of collinearity, diabetes mellitus, parity, pre-pregnancy BMI (Non-macrosomic infants).

Masukume G, Khashan AS, Morton SMB, Baker PN, Kenny LC, McCarthy FP. Caesarean section Delivery and Childhood Obesity in a British Longitudinal Cohort Study. *PLoS One*. 2019. DOI: 10.1371/journal.pone.0223856
