# Supplementary material for: Caesarean section delivery and childhood obesity in a British longitudinal cohort study
Source: PLoS One. 2019 Oct 30;14(10):e0223856. doi: 10.1371/journal.pone.0223856 (PMC6821069; doi:10.1371/journal.pone.0223856)
Supplement: S7 Table — (PDF) [file pone.0223856.s007.pdf]

S7 Table. Mode of birth and body mass index for male infants.

BMI		Coef (95% CI)	p-value	AdjCoef (95% CI)**	p-value
	Normal vaginal	reference		reference	
	Assisted vaginal	-0.05 (-0.18; 0.08)	0.410	-0.02 (-0.15; 0.11)	0.721
	Planned Caesarean	0.29 (0.15; 0.43)	0.000	-0.08 (-0.06; 0.22)	0.282
	Emergency Caesarean	0.23 (0.11; 0.35)	0.000	0.10 (-0.03; 0.23)	0.131

Time points for adjusted model = 25,041. Mixed-effects linear regression. BMI – Body mass index, Coef (Coefficient), CI (Confidence intervals), Adj (Adjusted).
**Adjusted for maternal age, ethnicity, education, marital status, couple income, infant sex – omitted because of collinearity, birth weight, smoking, gestational age, diabetes mellitus, parity, pre-pregnancy BMI (Non-macrosomic infants).

Masukume G, Khashan AS, Morton SMB, Baker PN, Kenny LC, McCarthy FP. Caesarean section Delivery and Childhood Obesity in a British Longitudinal Cohort Study. *PLoS One*. 2019. DOI: 10.1371/journal.pone.0223856
