# Supplementary material for: Caesarean section delivery and childhood obesity in a British longitudinal cohort study
Source: PLoS One. 2019 Oct 30;14(10):e0223856. doi: 10.1371/journal.pone.0223856 (PMC6821069; doi:10.1371/journal.pone.0223856)
Supplement: S8 Table — (PDF) [file pone.0223856.s008.pdf]

S8 Table. Mode of birth and body mass index for female infants.

BMI		Coef (95% CI)	p-value	AdjCoef (95% CI)**	p-value
	Normal vaginal	reference		reference	
	Assisted vaginal	-0.08 (-0.23; 0.07)	0.291	-0.22 (-0.15; 0.11)	0.721
	Planned Caesarean	0.09 (-0.06; 0.23)	0.237	-0.09 (-0.06; 0.22)	0.282
	Emergency Caesarean	0.16 (0.02; 0.29)	0.024	0.10 (-0.03; 0.23)	0.131

Time points for adjusted model = 25,041. Mixed-effects linear regression. BMI – Body mass index, Coef (Coefficient), CI (Confidence intervals), Adj (Adjusted).
**Adjusted for maternal age, ethnicity, education, marital status, couple income, infant sex – omitted because of collinearity, birth weight, smoking, gestational age, diabetes mellitus, parity, pre-pregnancy BMI (Non-macrosomic infants).

Masukume G, Khashan AS, Morton SMB, Baker PN, Kenny LC, McCarthy FP. Caesarean section Delivery and Childhood Obesity in a British Longitudinal Cohort Study. *PLoS One*. 2019. DOI: 10.1371/journal.pone.0223856
