# Supplementary figures and images for: Caesarean section delivery and childhood obesity in a British longitudinal cohort study
Source: PLoS One. 2019 Oct 30;14(10):e0223856. doi: 10.1371/journal.pone.0223856 (PMC6821069; doi:10.1371/journal.pone.0223856)

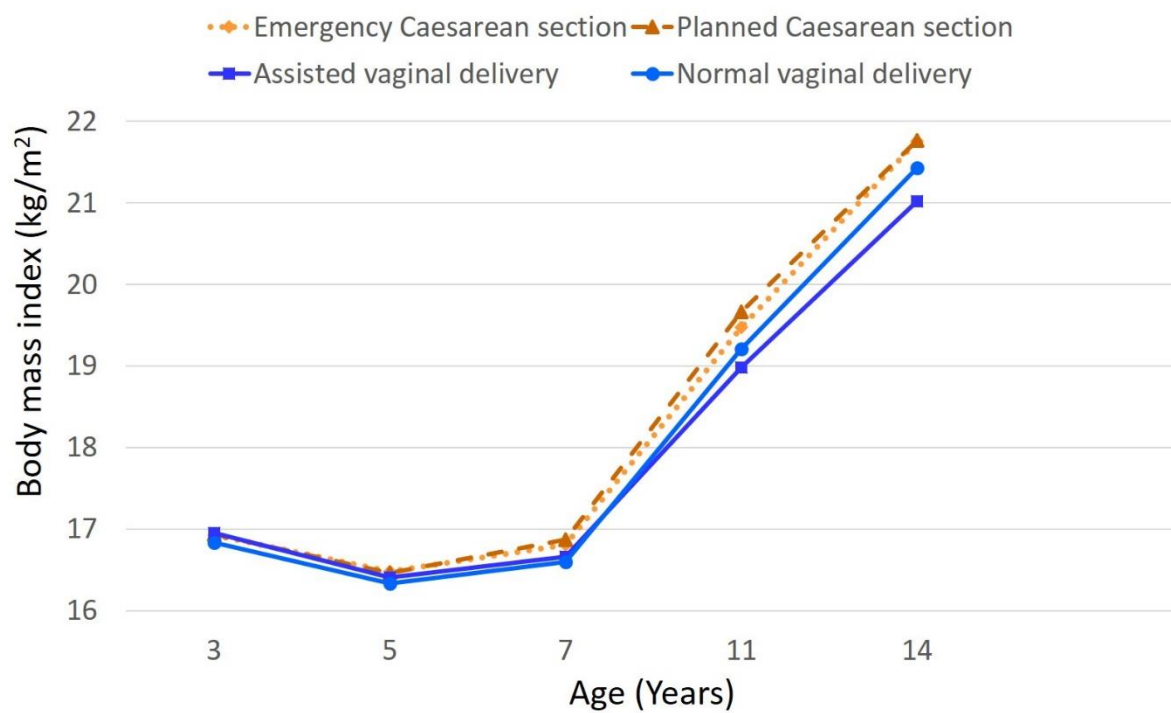

**S1 Fig. Mean body mass index by birth mode from age three to fourteen years.**

Supplement: S1 Fig — (PDF) [file pone.0223856.s009.pdf]
