# Supplementary material for: Caesarean section delivery and childhood obesity in a British longitudinal cohort study
Source: PLoS One. 2019 Oct 30;14(10):e0223856. doi: 10.1371/journal.pone.0223856 (PMC6821069; doi:10.1371/journal.pone.0223856)
Supplement: Attachment — Submitted filename: Response to Reviewers.docx [file pone.0223856.s010.docx]

The Irish Centre for Fetal and Neonatal Translational Research (INFANT)

5th Floor Cork University Maternity Hospital

Wilton, Cork, Republic of Ireland

+353 830409737

[Fergus.mccarthy@ucc.ie](mailto:Fergus.mccarthy@ucc.ie)

18th August 2019

The Editor

*PLOS ONE*

Dear Editor,

**RE: PONE-D-19-17591 Caesarean Section Delivery and Childhood Obesity in a British Longitudinal Cohort Study**

Thank you for sending our manuscript for review and for now considering it for publication. We are grateful to the peer reviewers for their helpful comments which strengthen our manuscript. We respond point-by-point to each comment below and have made changes where appropriate. We have highlighted our changes in the revised manuscript.

**Academic Editor’s comments**

Please address all the Reviewers' remarks in your revised manuscript, with a special attention to the points made by Reviewer 1 regarding the definitions of weight categories, and the methodology of the study.

We have given special attention to the points made by Reviewer 1.

In addition, we feel that some important, more subtle markers of metabolic health in and around pregnancy are missing as potentially confounding factors, such as weight gain during pregnancy and post-pregnancy weight retention, and would suggest their addition, would they be available.

These are indeed important considerations. We have thus added:

“The confounding factor maternal gestational weight gain, which is linked to post-pregnancy weight retention, was not available. This limited our study. However because of the high degree of correlation between pre-pregnancy BMI and gestational weight gain we believe our models had sufficient merit.[1, 2]”

Page 12, Line 282

**Reviewer #1:**

This paper brings significant information about the impact of delivery on later risk of obesity. The cohort and statistical study are well designed.

Thank you for your kind comment.

However, the criteria used to define weight categories should be updated since there is a risk of misclassification:
The IOTF criteria rely on 6 populations sample around the world but do not represent the growth pattern of a real child. Nowadays, WHO reference curves are much better tools which can be used in both clinical and epidemiological settings. They should be used instead since they reflect actual growth patterns.

We apologise for not communicating clearly what our main analysis was and where and how we used the IOTF classification. We have now sought to communicate these aspects more clearly:

Our main analysis was based on BMI as a continous measure, independent of a BMI classification system. We have now clarified this:

“In the main analysis, to account for the continuous BMI, repeated measures available at age three, five, seven, eleven and fourteen years, crude and adjusted mixed-effects linear regression models were generated.”

Page 6, Line 140

The IOTF criteria were used only to calculate the prevalence of obesity and to replicate a specific portion of our prior work. We have now clarified this as follows:

“Childhood obesity prevalence, at age three, five, seven, eleven and fourteen years was calculated using the International Obesity Taskforce criteria.”

Page 2, Line 37

“In secondary analysis, to replicate our prior work,[3] multinomial logistic regression models were fitted to investigate the association between birth mode and IOTF BMI category transition between age three and five years; 0 = remained normal (base outcome), 1 = remained obese, 2 = became obese, 3 = became non-obese and 4 = any other transition.”

Page 6, Line 142

Some like Kêkê and colleagues have suggested that the IOTF criteria have the strength of being best adapted for population studies.[4] Despite this, there are differences of opinion amongst researchers in this field, and an alternative is use of the WHO criteria.

We have now calculated the WHO z-scores where applicable:

“According to the WHO criteria overweight and obesity prevalence at age three years was 5.2% and 1.8% respectively (Supplementary Table S1).”

Page 8, Line 173

We have updated the manuscript as follows:

“Of the major BMI classification systems, including those from the World Health Organization (WHO) and Centers for Disease Control and Prevention (CDC), the IOTF criteria have been the most frequently used for this research topic.[4, 5] Using the 2006 WHO child growth standards, anthropometric z-scores were also calculated.[6]”

Page 6, Line 131

We acknowledge that there was concern in the scientific community around the IOTF criteria because there could not be expressed as centiles like the WHO references. In addition to the landmark IOTF papers, that we had cited, we have now cited the 2012 Extended IOTF cut-offs where centiles were derived.[7]

Reference

36.	Cole TJ, Lobstein T. Extended international (IOTF) body mass index cut-offs for thinness, overweight and obesity. Pediatric obesity. 2012;7(4):284-94. Epub 2012/06/21. doi: 10.1111/j.2047-6310.2012.00064.x. PubMed PMID: 22715120.

Our preference remains the use of IOTF criteria, but we have now included data using the WHO criteria. We leave it to the reviewer’s and the editor’s discretion as to whether this is also included.

Another point is the use of bioimpedance in order to measure body composition: a recent study by Tanita reports a mean difference in body composition with DEXA of -6.75 % with what seems to be one of more recent device of this company. BIA is not considered a valid tool in paediatrics. This limit should be pinpointed. This should lead to the conclusion that BMI alone may be a sufficient tool in the field of epidemiology in paediatrics.

The reviewer makes a valid point that bioimpedance has limitations which should be pinpointed. We have inserted the following:

“Using bioelectric impedance, for large studies like the MCS, is advantageous because of its portability, ease of use and low cost; the disadvantage however is that BF% is underestimated.[8]”

Page 12, Line 284

The impact of puberty on the older category (14 years) is not quoted at all, albeit certain.
Puberty is indeed an important aspect that needs to be quoted. We have done so:

“Around the time of puberty,[9] an acceleration of BMI towards adult values was observed at age eleven and fourteen years, however the association between delivery mode and BMI remained non-significant.”

Page 11, Line 256

Although using other BMI classification would not change the results of the comparisons of the absolute values of the BMI, it may bring different information about the shiftes form categories of BMI or the proportions of children in each category.
The strength of this paper would be very much improved using up to date paediatric tools.

We are in agreement with the reviewer’s statement.

We have now included:

“Using other BMI classification, like the WHO system, would not change the results of the comparisons of the absolute values of BMI.”

Page 12, Line 288

As above, we have calculated proportions based on the WHO system.

We hope that our clarification, above, of the tools that we used demonstrates that they are up to date paediatric tools.

**Reviewer #2:**

This is a well written paper that clearly motivates the contribution it makes over the prior literature. It is also an important topic on which to publish null findings. The data they use is well suited for testing their hypothesis. They also have an excellent description of the proposed mechanisms. The analysis is well done, including multiple imputation for missing data, and I have only a few minor comments/suggestions.

Thank you for your kind comment.

1. On line 108, do the authors mean “accurate” rather than “precise”?

We meant accurate and have now corrected this.

Page 5, Line 112

2. It would be useful in the introduction to discuss the estimates of association with obesity published in the literature, ideally from the meta-analyses, so the adequacy of power to detect a null hypothesis could be assessed. That is, is the sample size here enough to detect the non-null coefficients that are presumably biased?

In the introduction we have now stated:

“According to the systematic reviews and meta-analyses estimates of the strength of association between birth mode and childhood obesity, albeit with bias favouring positive effects, have been generally less than a relative risk of 1.50.[5, 10]”

Page 5, Line 104

With respect to power, to the limitations section we have added:

“Although there was inherent lack of power for some analyses, particularly at later ages because of loss to follow-up, consistency of the results suggests their merit.”

Page 13, Line 298

3. The separation of planned and emergency CS is very innovative. I think the authors could highlight more specifically the fact that these two procedures likely have different confounding structures, thus the fact that results are null for both types of exposures adds additional credibility to their null findings.

We agree that the separation of planned and emergency CS is very innovative. We agree that these two procedures likely have different confounding structures. Although the results were null for both types of exposure, the point estimates were generally greater for emergency CS than for planned CS indicative of a different underlying confounding structure. To the manuscript we have thus added:

“The exposures planned and emergency CS likely have different confounding structures. Although the results were null for both types of exposure, the point estimates were generally greater for emergency CS than for planned CS reflective of this underlying dissimilar confounding structure.”

Page 11, Line 253

4. Are there any further variables to capture the health of the mother prior to birth that could be controlled for? I think the list is adequate, but in particular curious about indicators of poor health that would predict emergency C-section. Also, given critical importance of maternal pre-pregnancy BMI as a confounder, how was this controlled for? It should be used in a way that accounts for non-linearity of confounding.

Multiple imputation was used to control for missing data from maternal pre-pregnancy BMI, which was included in the models.

Unfortunately, there were no further variables to capture the health of the mother prior to birth. We have expanded the limitations as follows:

“It was not possible to rule out possible confounding due to the underlying reasons for CS because there were no further variables like previous CS available to capture the health of the mother prior to birth and the exact indications for CS birth were unavailable.”

Page 12, Line 292

We hope that we have provided sufficient clarification. We look forward to your consideration.

Kindest regards,

Gwinyai Masukume

MB ChB, Dip Obst, MSc

On behalf of all Authors

**References**

1.	Bogaerts A, De Baetselier E, Ameye L, Dilles T, Van Rompaey B, Devlieger R. Postpartum weight trajectories in overweight and lean women. Midwifery. 49:134-41. doi: 10.1016/j.midw.2016.08.010.

2.	Heslehurst N, Vieira R, Akhter Z, Bailey H, Slack E, Ngongalah L, et al. The association between maternal body mass index and child obesity: A systematic review and meta-analysis. PLoS medicine. 2019;16(6):e1002817. Epub 2019/06/12. doi: 10.1371/journal.pmed.1002817. PubMed PMID: 31185012; PubMed Central PMCID: PMCPMC6559702.

3.	Masukume G, O'Neill SM, Baker PN, Kenny LC, Morton SMB, Khashan AS. The Impact of Caesarean Section on the Risk of Childhood Overweight and Obesity: New Evidence from a Contemporary Cohort Study. Scientific reports. 2018;8(1):15113. Epub 2018/10/13. doi: 10.1038/s41598-018-33482-z. PubMed PMID: 30310162.

4.	Kêkê LM, Samouda H, Jacobs J, di Pompeo C, Lemdani M, Hubert H, et al. Body mass index and childhood obesity classification systems: A comparison of the French, International Obesity Task Force (IOTF) and World Health Organization (WHO) references. Revue d'Épidémiologie et de Santé Publique. 2015;63(3):173-82. doi: <https://doi.org/10.1016/j.respe.2014.11.003>.

5.	Kuhle S, Tong OS, Woolcott CG. Association between caesarean section and childhood obesity: a systematic review and meta-analysis. Obesity reviews : an official journal of the International Association for the Study of Obesity. 2015;16(4):295-303. Epub 2015/03/11. doi: 10.1111/obr.12267. PubMed PMID: 25752886.

6.	Leroy J. zscore06: Stata command for the calculation of anthropometric z-scores using the 2006 WHO child growth standards. 2011.

7.	Cole TJ, Lobstein T. Extended international (IOTF) body mass index cut-offs for thinness, overweight and obesity. Pediatric obesity. 2012;7(4):284-94. Epub 2012/06/21. doi: 10.1111/j.2047-6310.2012.00064.x. PubMed PMID: 22715120.

8.	Delisle Nystrom C, Henriksson P, Alexandrou C, Lof M. The Tanita SC-240 to Assess Body Composition in Pre-School Children: An Evaluation against the Three Component Model. Nutrients. 2016;8(6). Epub 2016/06/21. doi: 10.3390/nu8060371. PubMed PMID: 27322313; PubMed Central PMCID: PMCPMC4924212.

9.	Alotaibi MF. Physiology of puberty in boys and girls and pathological disorders affecting its onset. J Adolesc. 2019;71:63-71. Epub 2019/01/15. doi: 10.1016/j.adolescence.2018.12.007. PubMed PMID: 30639665.

10.	Sutharsan R, Mannan M, Doi SA, Mamun AA. Caesarean delivery and the risk of offspring overweight and obesity over the life course: a systematic review and bias-adjusted meta-analysis. Clinical obesity. 2015;5(6):293-301. Epub 2015/08/20. doi: 10.1111/cob.12114. PubMed PMID: 26286021.
